# Shifts in UNAIDS ethics guidance and implications for ethics review of preventive HIV vaccine trials

**DOI:** 10.1002/jia2.25796

**Published:** 2021-11-21

**Authors:** Catherine Slack, Paul Ndebele, Mary Allen, Jessica Salzwedel

**Affiliations:** ^1^ HIV/AIDS Vaccines Ethics Group (HAVEG) School of Applied Human Sciences College of Humanities University of KwaZulu‐Natal Pietermaritzburg South Africa; ^2^ Department of Global Health MilkenInstitute School of Public Health The George Washington University Washington DC USA; ^3^ National Institute of Allergy and Infectious Diseases National Institutes of Health Bethesda Maryland USA; ^4^ AIDS Vaccine Advocacy Coalition (AVAC) New York USA

**Keywords:** ethics, HIV vaccine trials, REC, UNAIDS

## Abstract

**Introduction:**

A major change in the ethics framework for preventive HIV vaccine trials worldwide is the release of the UNAIDS 2021 *ethical considerations in HIV prevention trials*. This new guidance comes at an exciting time when there are multiple HIV vaccine efficacy trials in the field. Research Ethics Committees (RECs) or Institutional Review Boards are a most likely audience for these guidelines. Our objective is to highlight shifts in ethics recommendations from the earlier 2012 UNAIDS guidance.

**Discussion:**

We review recommendations related to four key issues, namely standard of prevention, post‐trial access to safe and effective vaccines, enrolment of adolescents and enrolment of pregnant women. We outline implications and make recommendations for the ethics review process, including suggested lines of inquiry by RECs and responses by applicants.

**Conclusions:**

There have been several shifts in the UNAIDS ethics guidance with implications for HIV vaccine researchers submitting applications for initial ethics review or re‐certification, *and* for RECs conducting such reviews. This review may assist RECs in a more efficient and consistent application of ethics recommendations. However, additional tools and training may further help stakeholders comply with new UNAIDS ethics recommendations during protocol development and ethics review.

## 1 INTRODUCTION

Currently, three preventive HIV vaccine efficacy trials are underway – HVTN 705 and 706 are exploring the Janssen HIV vaccine regimen in adults [1] and an additional trial – PrEPVacc – has begun in Africa to evaluate two DNA‐based regimens in combination with oral pre‐exposure prophylaxis (PrEP) [2]. Other vaccine regimens are in earlier phase trials [1]. It may be several years before other preventive HIV vaccine candidates progress to efficacy trials. Such late‐phase trials, enrolling populations at high‐risk, require Research Ethics Committees (RECs) or Institutional Review Boards to carefully attend to plans for participants’ access to HIV prevention tools, plans for post‐trial access to vaccines proven safe and effective, as well as plans for enrolment of pregnant women, and enrolment of adolescents.

While not specific to review of HIV vaccine trials, it has been argued that REC review processes can be inefficient or demonstrate unjustified variations across RECs during review of multi‐site trials [3]. This may be due in part to REC review capacity when faced with complex protocols, as well as uneven application of ethics guidance. Also, the expertise with which researchers/applicants pro‐actively address ethics issues impacts the efficiency and quality of ethics review [4, 5]. Centralized ethics review and reciprocal review models (or reliance agreements) are used in some settings to increase efficiency and harmony of REC review; however, in many settings, ethical‐legal frameworks and institutional policy lead to multi‐REC review.

A major development in the ethics framework for preventive HIV vaccine trials is the release of the new *Ethical Considerations for HIV Prevention Trials* (UNAIDS, 2021) with the expressed hope that these will be a “valuable resource for RECs” [6] and other key stakeholders. While some changes have been summarized [6], there has been little effort to consider how shifts in guidance might (or should) impact RECs and researchers in the ongoing ethics review process; our objective is to describe these shifts. Given that RECs and researchers have been exposed to UNAIDS recommendations for 13 years [7], we do not present recommendations in their entirety here, rather we focus on *shifts* in this guidance. Also, we focus on trials of preventive HIV vaccines, even while trials of other HIV prevention products are underway in order to expand the range of options for all at‐risk populations [8, 9, 10].

## 2 DISCUSSION

### 2.1 Standard of prevention

As highly effective oral PrEP has become increasingly available, the “welcome but difficult” challenge of reduced incidence in efficacy trial design has been underscored [11]. Long‐acting PrEP, which includes injectables and vaginal rings, may also become progressively more available [9] as effective products receive regulatory approval and novel drugs progress through the pipeline. HIV vaccine trial designs need to balance ensuring participants’ access to prevention methods while simultaneously supporting efficacy assessments through sufficiently powered studies [9]. How to best achieve this balance has been the subject of intense discussion and debate [12].

The UNAIDS 2021 [13] revision is expressly rooted in the above dilemma, namely the need to research future interventions to address the unmet needs of at‐risk populations, while simultaneously facilitating access to effective interventions for current participants. A marked change in the 2021 recommendations is that researchers should ensure that participants get access to “*WHO‐recommended*” prevention methods, as opposed to “*state of the art*” prevention [7] – the latter being criticized as “infeasible in practice” [14]. Another change lies in *explicit* guidance that researchers can deviate from the “WHO‐recommended” standard only for compelling scientific, biological or manufacturing reasons; and that relevant stakeholders must be meaningfully engaged in review of the deviation, and their acceptance of the deviation is required. This replaces *implied* guidance that researchers could (presumably) deviate from the “*state of the art*” standard after consultation with stakeholders taking into account feasibility, impact and ability to isolate the modality being tested. Another shift is that deviations from WHO‐recommended prevention methods must be approved by RECs, which makes RECs central to decisions about acceptable prevention standards, as encouraged by previous scholars [15].

In terms of review of *standard of prevention*, RECs may establish a requirement for local applicants to demonstrate that the “WHO‐recommended” standard is met for participants *at their site*, with support from networks where possible, for example, in a participant HIV prevention plan. Networks (or pharmaceutical companies) could implement engagement efforts at an international level to debate justified deviations (to decrease pressure on individual sites to achieve such engagement goals), and describe these engagement efforts in master protocols. Sites could be encouraged to develop site‐specific descriptions of their local engagement efforts. RECs can conduct an efficient three‐part assessment: 1) Has the standard been met? 2) If not, is the deviation justified? 3) Have engagement efforts been “meaningful”? Here, RECs can ask insightful questions regarding the quality of engagement, for example about the inclusiveness and diversity of engagement [16].

### 2.2 Post trial access

Commentators have noted that “the possibility of an effective (HIV) vaccine is now tangible” [11]. COVID‐19 provides sobering lessons about post‐trial access to vaccines [17], including that low‐ and middle‐income countries which take part in trials but cannot make early monetary investments will likely lag behind those countries that can invest substantially in developing and manufacturing vaccines, and can negotiate purchasing well before clinical trials and manufacturing are complete [18]. COVID‐19 also underscores the need to invest in infrastructure to deliver vaccines with demanding cold‐chain requirements [19, 20].

A significant shift in the UNAIDS 2021 [13] guidance is that plans for access should be in the protocol. In terms of *non‐participants* having access to products shown in the clinical trial to be safe and effective, the 2021 guidance reiterates earlier recommendations for sponsors and researchers to initiate discussions as well as secure an agreed plan regarding how products will be approved, paid for, manufactured and delivered/distributed, while introducing that plans considers the potential complexities of the pathway. Like its predecessor, the 2021 guidance recommends that sponsors and researchers cast the net widely for planning and discussions but 2021 guidance encourages for the first time “special attention” to populations often excluded from research and product access. In terms of access to products *by participants*, the former document made a procedural recommendation that sponsors *secure agreement* on plans to make the intervention available to participants, while 2021 guidance makes a substantive recommendation that sponsors should ensure “ongoing provision” and “continued access” to participants. It is also more nuanced than its predecessor insofar as it provides reasons for deviating from this stance. In terms of access to results, 2021 guidance introduces that *participants, local communities and national governments* should receive results “before or contemporaneously with international dissemination”.

In terms of review of *access to products*, RECs can require protocol submissions to include statements that show planning for access to proven products (and to results) for key groups like participants, the community engaged in the trial or population in which the product was tested. In phase 1/2 protocols, RECs can inquire about potential subsequent trials as well as broad plans for access, and prompt sponsor/investigators to provide descriptions about issues, such as cost, and capacity needed to produce and deliver the product. Such inquiries relatively early on may help trigger actions with long time‐frames, such as facility development to manufacture vaccines, or manage the cold‐chain. In phase 3 protocols, RECs should expect more detailed planning during initial review and make regular inquiries about progress on this issue during annual reports/re‐certifications. RECs can address any concerns about access to products being an “undue” inducement by ensuring careful framing in consent materials, and ensuring that study risks are not minimized or discounted [21].

### 2.3 Enrolment of adolescents

Adolescents are at high risk of HIV infection worldwide because of various behavioural, biological and structural factors; however, their inclusion in trials is made complex by several elements, including regulatory frameworks [22, 23]. The 2021 guidance no longer has a guidance point specifically devoted to adolescents; instead, recommendations specifically relating to adolescents are included largely under the “fair selection” guidance point, with additional remarks under “informed consent” and “confidentiality and privacy”. Hence, users have to work harder to locate scattered recommendations.

In terms of adolescent‐specific guidance, the 2021 document retains the 2012 recommendation to seek parental consent “unless exceptions are authorized by national legislation” but somewhat softens the stance by permitting exceptions authorized in “national guidelines”. The guidance now acknowledges that parental consent for sensitive research can act as a “barrier” to enrolment; can cause “social harms” like parental sanctions; and can “skew enrolment” towards low‐risk adolescents. In terms of adolescent‐relevant guidance, a subtle but striking shift is that the 2021 document squarely faces the problem of under‐representation of key groups in trials leading to health disparities. The revised guidance repeatedly encourages researchers to avoid arbitrarily excluding persons, that is without good scientific reasons, justifications or goals. REC members will rightly remember several 2012 recommendations along these lines, but less emphasis on the *problem* of an inequitable evidence base for under‐represented groups. Also, the 2021 document attempts to shift away from vulnerable persons towards *contexts in which people live* that increase their vulnerability [6] while retaining most of the recommended responses from the earlier document. Another shift in the 2021 guidance is that researchers are encouraged to recognize gender diversity; to include gender‐diverse groups in trials; and to attend to the needs of individuals of all gender identities and expressions.

The 2021 guidance emphasis on the problematic under‐representation of *adolescents* means RECs have a substantial basis for making inquiries about plans to enrol adolescents. Because of shifts away from vulnerable people to *contexts of vulnerability*, RECs should be alert for descriptions about contexts that may increase vulnerabilities to harms, for example laws that criminalize same‐sex behaviour or under‐age consensual sexual activity among adolescent peers [24]. A shift to highlight gender diversity paves the way for RECs to make insightful inquiries about plans to reach gender‐diverse adolescents, and even inquire about prior training on such matters for study staff. Subtle shifts in consent language mean that RECs should be more aware of the potential consequences of parental consent strategies. The acceptability of parental waivers in some contexts depends on whether interventions present an acceptable level of risk [23], and risks of HIV preventive vaccine trials in adolescents (including social impacts from Vaccine Induced Sero‐Positivity [25]) will require careful attention by RECs when considering the appropriate consent approach to adolescent enrolment.

### 2.4 Enrolment of pregnant women

Some have argued that HIV preventive trials involving pregnant women lag behind HIV treatment trials [26]. However, only one HIV vaccine regimen has shown even modest efficacy [27] that did not justify testing the regimen during pregnancy. This is in contrast to HIV treatment trials where several regimens have demonstrated sufficient safety and efficacy in non‐pregnant women to justify enrolment of pregnant women. A recent review encourages guideline‐developers (among others) to “affirm the imperative for responsible research with pregnant women” [28].

In a similar vein, UNAIDS 2021 [13] shifts to underscoring problematic evidence gaps and inequities in access for groups, including “pregnant women or gender diverse persons”, even while its predecessor opposed exclusion of vulnerable groups like pregnant women without scientific reasons. Like its predecessor, the 2021 document recommends timely discussion to resolve the exclusion of pregnant persons. But the 2021 document shifts the timing of such discussions *earlier* – when the candidate “has sufficient promise to advance into phase 2b or 3” – and removes the choice to time the discussion after the trial product has been demonstrated effective. Also, the 2021 guidance shifts the topic of discussion to “pregnancy‐specific pharmacokinetic (PK)” studies away from “safety studies”. These shifts resonate with recommendations for the early integration of pregnancy‐specific pharmacokinetic studies into development plans so that new interventions “reach the market with pregnancy‐specific dosing information” at or closely after licensure [28].

It is also important to explore whether experimental HIV vaccines administered to infants born to women living with HIV (in combination with known safe and effective antiretroviral treatment [ARTs]) can prevent transmission of HIV from the mother. For example, HVTN 135 is enrolling mother–infant pairs in South Africa, and is vaccinating infants. The UNAIDS 2021 guidance document asserts that trials to prevent mother‐to‐child transmission are outside the scope of the guidance, even while many guidance points remain relevant.

In terms of ethics review of *enrolment of pregnant women and gender diverse persons*, the shift towards an equitable evidence base for pregnant persons in UNAIDS 2021 means that RECs ought to “proactively work with investigators to identify approvable designs” [28]. It follows that RECs should not *generally* default to exclusion but adopt the “burden of careful analysis” of each protocol *individually* [29] to see if acceptable risk–benefit ratios are met. RECs are responsible for assessing whether the risks of study procedures/interventions are justified by either the potential for direct benefit to the pregnant person and/or the foetus, or by generalizable knowledge to other pregnant persons and foetuses and in the latter instance, no greater risk than “minimal harm” to the foetus is required [29]. Assessing if a favourable risk–benefit ratio can be met for each component of the trial in a component analysis can be a useful approach [30], and RECs should carefully document their inclusion/exclusion decisions [29]. In terms of persons who become pregnant while participating in trials, RECs should ensure all protocols address collection of relevant safety data from the pregnant participant, and data on the outcome of the pregnancy. In some protocols, collection of safety data on the newborn/infant may be justified. Having experts in maternal/foetal medicine on RECs may be an important action in supporting quality ethics review.

## 3 CONCLUSIONS

There have been several shifts in the UNAIDS ethics guidance with implications for HIV vaccine researchers submitting applications for initial review or re‐certifications, *and* for RECs conducting reviews. These shifts, made in response to contemporary challenges in the field, may not harmonize completely with other leading guidance; for example, UNAIDS 2021 standard of prevention is “WHO‐recommended”, whereas CIOMS 2016 recommends “established effective” and HPTN 2020 recommends “known effective”, “practically achievable”, and “reasonably accessible”. A thorough head‐to‐head comparative analysis is needed to identify where substantive differences are large on key concerns, and advocacy will be needed to resolve tensions that threaten the ethics evaluation of HIV vaccine studies. This paper may go some way towards indirectly improving efficiency and consistency in review processes [4, 31, 32]. However, additional tool development and training may further help affected stakeholders to recalibrate protocol development and ethics review in a way that reflects the impact of new UNAIDS ethics recommendations.

## COMPETING INTERESTS

CS serves on a DSMB that has oversight of certain HIV vaccine trials and DSMB members receive an honorarium from NIAID. She was part of the consultation hosted by WHO in the process of guideline revision.

PN serves on a Research Ethics Committee that has oversight of certain HIV vaccine trials.

MA is employed by the National Institutes of Health, a US government agency that has funded clinical trials of preventive HIV vaccines and monoclonal antibodies, and she has served as medical officer for such trials.

JS has no competing interests.

## AUTHORS' CONTRIBUTIONS

CS conceptualized the commentary, reviewed the guidance and developed the first draft. PN, MA and JS revised the article for important content.

## References

[1] National Institute of Health [NIH] . U.S. National Library of Medicine: ClinicalTrials.gov. 2021. Available from: https://clinicaltrials.gov/. Access 1 April 2021

[2] PREPVAC . PREPVAC. 2021. Available from: https://www.prepvacc.org/. Access 1 April 2021

[3] Abbott L , Grady C . A systematic review of the empirical literature evaluating IRBs: what we know and what we still need to learn. J Empir Res Hum Res Ethics. 2011;6(1):3–19. 10.1525/jer.2011.6.1.3 PMC3235475 21460582

[4] Silaigwana B , Wassenaar D . Biomedical Research Ethics Committees in sub‐Saharan Africa: a collective review of their structure, functioning, and outcomes. J Empir Res Hum Res Ethics. 2015;10(2):169–84. 25819759 10.1177/1556264615575511

[5] Silaigwana B , Wassenaar D . Research Ethics Committees’ oversight of biomedical research in South Africa: a thematic analysis of ethical issues raised during ethics review of non‐expedited protocols. J Empir Res Hum Res Ethics. 2019;14(2):107–16. 30678521 10.1177/1556264618824921

[6] van der Graaf R , Reis A , Godfrey‐Faussett P . Revised UNAIDS/WHO ethical guidance for HIV prevention trials. JAMA. 2021;325(17):1719–1720. 33502463 10.1001/jama.2021.0258

[7] UNAIDS, WHO . Ethical considerations in biomedical HIV prevention trials [additional guidance point added in 2012]. 2012.

[8] Brown BJ , Sugarman J . Why ethics guidance needs to be updated for contemporary HIV prevention research. J Int AIDS Soc. 2020;23(5):e25500. 32406990 10.1002/jia2.25500 PMC7224307

[9] Janes H , Zhu Y , Brown ER . Designing HIV vaccine efficacy trials in the context of highly effective non‐vaccine prevention modalities. Stat Biosci. 2020;12(3):468–94. 10.1007/s12561-020-09292-1 PMC10022814 36938334

[10] Sugarman J , Celum CL , Donnell D , Mayer KH . Ethical considerations for new HIV prevention trials. Lancet HIV. 2019;6(8):e489–91. 31221591 10.1016/S2352-3018(19)30184-5

[11] Bekker L‐G , Tatoud R , Dabis F , Feinberg M , Kaleebu P , Marovich M , et al. The complex challenges of HIV vaccine development require renewed and expanded global commitment. Lancet North Am Ed. 2020;395(10221):384–8. 10.1016/S0140-6736(19)32682-0 31806257

[12] Haire B , Folayan MO , Hankins C , Sugarman J , McCormack S , Ramjee G , et al. Ethical considerations in determining standard of prevention packages for HIV prevention trials: examining PrEP. Dev World Bioeth. 2013;13(2):87–94. 23725227 10.1111/dewb.12032 PMC3716830

[13] UNAIDS . Ethical considerations in HIV prevention trials. 2021.

[14] Brown BJ , Sugarman J ; HPTN Ethics Working Group . HPTN ethics guidance for research. 2020.

[15] Essack Z , Wassenaar DR . South African Research Ethics Committee review of standards of prevention in HIV vaccine trial protocols. J Empir Res Hum Res Ethics. 2018;13(3):239–46. 29631486 10.1177/1556264618763422

[16] Slack C , Wilkinson A , Salzwedel J , Ndebele P . Strengthening stakeholder engagement through ethics review in biomedical HIV prevention trials: opportunities and complexities. J Int AIDS Soc. 2018;21:e25172. 30334604 10.1002/jia2.25172 PMC6193317

[17] del Rio C , Gonsalves G , Hassan F , Kavanagh M . The BMJ opinion [Internet]. BMJ Publishing Group Limited. 2021. [cited 2021]. Available from: https://blogs.bmj.com/bmj/2021/03/17/covid‐19‐a‐call‐for‐global‐vaccine‐equity/.

[18] Duke Global Health Innovation Centre . Launch and Scale Speedometer. 2020.

[19] Hussain W . COVID‐19 vaccination challenges in developing countries: COVID‐19 vaccination challenges. Int J Front Sci. 2021;5(1):1–2.

[20] World Health Organization [WHO] . COVID‐19 vaccination: supply and logistics guidance. 2021.

[21] Emanuel E . Ending concerns about undue inducement. J Law Med Ethics. 2004;32:100–5. 10.1111/j.1748-720x.2004.tb00453.x 15152431

[22] Day S , Kapogiannis BG , Shah SK , Wilson EC , Ruel TD , Conserve DF , et al. Adolescent participation in HIV research: consortium experience in low and middle‐income countries and scoping review. Lancet HIV. 2020;7(12):e844–52. 33275917 10.1016/S2352-3018(20)30269-1 PMC8491773

[23] Knopf AS , Gilbert AL , Zimet GD , Kapogiannis BG , Hosek SG , Fortenberry JD , et al. Moral conflict and competing duties in the initiation of a biomedical HIV prevention trial with minor adolescents. AJOB Empir Bioeth. 2017;8(3):145–52. 28949893 10.1080/23294515.2016.1251506 PMC5618718

[24] Strode A , Singh P , Slack C , Wassenaar D . Research Ethics Committees in a tight spot: approving consent strategies for child research that are prima facie illegal but are ethical in terms of national guidelines. S Afr Med J. 2018;108(10):828–32. 30421709 10.7196/SAMJ.2018.v108i10.13203 PMC6237194

[25] Voronin Y , Zinszner H , Karg C , Brooks K , Coombs R , Hural J , et al. HIV vaccine‐induced sero‐reactivity: a challenge for trial participants, researchers, and physicians. Vaccine. 2015;33(10):1243–9. 25649349 10.1016/j.vaccine.2014.10.040 PMC4331207

[26] Wickremsinhe MN , Little MO , Carter AS , Sullivan KA , Lyerly AD . Beyond “vessels and vectors”: a global review of registered HIV‐related clinical trials with pregnant women. J Women's Health. 2019;28(1):93–9. 10.1089/jwh.2017.6857 PMC6343191 30124366

[27] Rerks‐Ngarm S , Pitisuttithum P , Nitayaphan S , Kaewkungwal J , Chiu J , Paris R , et al. Vaccination with ALVAC and AIDSVAX to prevent HIV‐1 infection in Thailand. N Engl J Med. 2009;361(23):2209–20. 19843557 10.1056/NEJMoa0908492

[28] The PHASES Working Group . Ending the evidence gap for pregnant women around HIV & co‐infections: a call to action. Chapel Hill; 2020.

[29] Payne P . Including pregnant women in clinical research: practical guidance for Institutional Review Boards. Ethics Hum Res. 2019;41(6):35–40. 10.1002/eahr.500036 31743630

[30] Weijer C . The ethical analysis of risk. J Law Med Ethics. 2000;28(4):344–61. 11317427 10.1111/j.1748-720x.2000.tb00686.x

[31] Ndebele P , Blanchard‐Horan C , Shahkolahi A , Sanne I . Regulatory challenges associated with conducting multi‐country clinical trials in resource‐limited settings. J Acquir Immune Defic Syndr. 2014;65(01):S29. 24321981 10.1097/QAI.0000000000000037 PMC3900239

[32] Nyika A , Kilama W , Chilengi R , Tangwa G , Tindana P , Ndebele P , et al. Composition, training needs and independence of ethics review committees across Africa: are the gate‐keepers rising to the emerging challenges? J Med Ethics. 2009;35(3):189–93. 19251972 10.1136/jme.2008.025189 PMC2643018

